# Elucidating Defect Behaviors Optimizing the Thermoelectric Performance in PbTe–MgTe Based Materials

**DOI:** 10.3390/ma19132809

**Published:** 2026-07-02

**Authors:** Xuemei Zhang, Jinwu Zhang, Mi Qin, Lulu Huang

**Affiliations:** 1 School of Physics and Electronic Information Engineering, Ningxia Normal University, Guyuan 756000, China; jwzhang@nxnu.edu.cn; 2 School of Microelectronics, Wuhan Textile University, Wuhan 430200, China; 3 School of Materials Science and Engineering, Hefei University of Technology, Hefei 230009, China

**Keywords:** thermoelectrics, point defect, PbMgTe solid solution, Seebeck coefficient

## Abstract

**Highlights:**

- Intrinsic defects (V_Pb_, V_Mg_, V_Te_, and Mg_i_) in PbMgTe solid solution have low formation energies and lead to p-type and n-type conductivity.
- The defect-induced electronic structure changes increase the density of states, significantly raising the Seebeck coefficient.
- High-throughput calculations reveal that defect behaviors critically limit or enhance PbMgTe thermoelectric performance.
- These insights guide defect engineering to narrow the performance gap between n-type and p-type PbMgTe materials.

**Abstract:**

PbTe–MgTe based compounds have been demonstrated as promising medium-temperature thermoelectric materials, and significant research efforts have been devoted to enhancing their performance. However, previous studies have primarily focused on low MgTe concentrations (within the solubility limit of ~6 mol%), and a systematic understanding of intrinsic defect behaviors in the PbMgTe solid solution remains lacking. In this work, we perform high-throughput density functional theory calculations to systematically evaluate a comprehensive set of intrinsic defects (including vacancies, anti-sites, and interstitials) in the PbMgTe solid solution modeled by SQS. To the best of our knowledge, this is the first systematic defect study in the PbMgTe system at this composition. Our calculations reveal that vacancies (V_Pb_, V_Mg_, V_Te_) and Mg interstitials (Mg_i_) exhibit low formation energies, with acceptor and donor behaviors that effectively facilitate p-type and n-type conductivity, respectively. Notably, these defects induce modifications in the electronic structure that lead to a significant enhancement of the density of states (DOS) near the band edges. Consequently, the Seebeck coefficient is markedly improved compared to that of intrinsic PbMgTe. Our work not only provides valuable insights for defect engineering in PbMgTe-based materials but also establishes a mechanistic link between defect-induced DOS changes and thermopower enhancement, advancing beyond previous studies that focused primarily on formation energies. These findings help bridge the performance gap between n-type and p-type thermoelectric properties.

## 1. Introduction

Thermoelectric materials, which enable the direct conversion of heat into electricity, have garnered significant attention as promising alternatives in the energy [1,2]. Thermoelectric performance can be characterized by the figure of merit, $zT=S^{2}\sigma T/\kappa$, where S, σ, T, and κ are the Seebeck coefficient, electrical conductivity, absolute temperature, and thermal conductivity, respectively [3,4,5,6]. To enhance the zT values, various strategies have been employed, among which defect engineering is particularly effective. For example, in doping or forming solid solutions, the band structures of the host can be significantly modified according to the band convergence [7,8], resonant levels [9,10,11,12], and band flatting [13,14]. These contribute to an improved power factor (PF = S^2^σ). Furthermore, the introduced dopants and alloying elements act as phonon scattering centers, effectively suppressing the lattice thermal conductivity by scattering phonons across different wavelengths: point defects scatter short-wavelength phonons, nanoscale precipitates medium-wavelength phonons, and grain boundaries scatter long-wavelength phonons [15]. Therefore, this synergistic approach to independently optimizing the electronic and thermal transport properties has been the driving force behind the progressive rise in reported zT values for state-of-the-art thermoelectric materials.

Among all thermoelectric materials, lead telluride (PbTe) stands as a cornerstone material that has been systematically studied for many years. It has established itself as a premier choice for applications in the medium-temperature regime (500–800 K), a status consistently supported by its high efficiency and extensive research history [16,17]. Jia et al. reports a peak zT value up to 2.8 at 850 K and an average zT value of 1.65 at 300 to 850 K in Pb_0.97_Na_0.03_Te–2%MgTe–75%GeTe, in which the energy conversion efficiency is ~15.5% at a temperature difference of 554 K in a segmented module [16]. To improve the thermoelectric properties of PbTe, the isoelectron and isostructured MTe (M = Zn, Sr, etc.) compounds have been introduced into the PbTe matrix to form solid solution and nanoscale precipitates when the MTe concentration is below and above the thermodynamic solubility limit, respectively. A record high ZT_avg_ of ∼1.26 for n-type Pb_0.975_Ga_0.025_Te–x%ZnTe compounds at 400–873 K with a maximum zT value of 1.55 at 723 K, which is melted via vacuum followed by spark plasma sintering [17]. A similar enhancement in the Seebeck coefficient and zT value has been reported in several PbTe-based systems, including PbTe–CdTe [18], PbTe–MnTe [19,20]. This improvement is consistently attributed to the successful modulation of PbTe’s multiple valence bands. It is reported that incorporation of MgTe into PbTe serves favorable functions for optimizing thermoelectric performance: (1) with its solubility limit in PbTe, Mg alloying optimizes the valence band structure by bringing the L and Σ valence bands closer in energy, which promotes charge carrier injection; (2) once the solubility limit of Mg is exceeded, abundant endotaxial nanostructures form throughout the matrix. These nanostructures, combined with mesoscale microstructural features, effectively scatter phonons across all relevant length scales, leading to a significant reduction in lattice thermal conductivity. Most notably, Mg alloying also widens the energy gap between the conduction band (CBM) and the valence band (VBM), thereby suppressing bipolar thermal conductivity through band gap enlargement [21]. Thus, understanding the behavior of intrinsic defects in PbTe–MgTe based compounds is essential for enhancing their thermoelectric performance.

For semiconductors, understanding the intrinsic defects is the first step to tune the properties of semiconductors. Goyal et al. use different levels of theory to model point defects in PbTe, finding that HSE + SOC + G_0_W_0_ is the approach that can accurately capture the intrinsic conductivity type of PbTe as well as the measured charge carrier concentrations [22]. Bajaj et al. study the defect formation energies of various intrinsic point defects in PbTe using DFT in conjunction with the CALPHAD method, which is beneficial for tuning Fermi level, and further to optimize the TE properties [23]. Understanding the physical and chemical nature of these defects can control or optimize thermoelectric properties. By embedding MgTe in the PbTe matrix, it will form the solid solution when the MgTe concentration is below the solubility limit [24,25,26,27]. Even when the concentration is higher than that of the solubility limit, a large solid solution region will be formed around the PbTe/MgTe interfaces. Therefore, it is important to understand the intrinsic defect behaviors in the in PbTe–MgTe based materials. Huo et al. apply the first-principles studies on the defects of classical thermoelectric materials Xte (X = Ge, Sn, and Pb). They report that the formation energies of the defects in PbTe with relatively high stability, such as V_Te_, Te_Pb_, etc., depend sensitively on the chemical potentials of Pb, as well as the accuracy of the band gap, and those defects tend to induce weak n-type transport by default [28]. To the best of our knowledge, such theoretical studies on the nature of the defects in the MgTe doping in PbTe compounds are rare, especially in the solid solution compounds.

In this study, we conduct a systematic investigation into the influence of defects on the transport properties of PbTe–MgTe based thermoelectric (TE) materials using first-principles combining a high-throughput defect computation. The computed defect formation energies reveal that intrinsic Pb and Mg vacancies act as characteristic p-type defects, while Mg_i_ interstitials and Te vacancies emerge as the predominant donor-type defects. This finding aligns well with the experimentally observed p and n-type conduction behavior in these materials. Through detailed electronic structure calculations, we demonstrate that Mg interstitial sites and Te vacancies can induce pronounced resonant states within the conduction band. The presence of these states significantly enhances the n-type thermopower (S), thereby enabling it to approach the level of excellent transport performance. Notably, our results indicate that the thermopower values for systems containing V_Pb/M_g and Mg_i_/V_Te_ defect complexes are substantially higher than that of the pristine PbMgTe compound. Beyond electronic transport, these defects also contribute to increased phonon scattering, leading to a marked reduction in the lattice thermal conductivity. Furthermore, we elucidate the mechanisms by which various defect types modify the microscopic electronic environment. Moreover, defects can either promote electron localization or delocalization through distinct pathways, which in turn directly impacts their formation energies, stability, and mutual interactions. This comprehensive analysis not only provides valuable guidance for future experimental defect engineering strategies aimed at optimizing PbMgTe-based materials, but also paves a way towards bridging the performance gap between n-type and p-type thermoelectric properties, ultimately contributing to the development of more efficient TE devices.

## 2. Methodology

### 2.1. Density Functional Theory

First-principles calculations based on density functional theory (DFT) are conducted using the Vienna ab initio simulation package (VASP.5.4.4) with the projector augmented wave (PAW) method [29,30,31]. The exchange–correlation energy is described within the generalized gradient approximation (GGA) using the Perdew–Burke–Ernzerhof (PBE) functional [30]. A plane-wave energy cutoff of 450 eV is applied for primitive cell calculations, to ensure total energy convergence within 10^−5^ eV. Considering spin-orbit coupling (SOC) is essential in Pb-containing chalcogenides, we also perform SOC in GGA-PBE calculation to correct the band gap for PbMgTe compounds. The Brillouin zone of the PbMgTe is sampled using a 7 × 7 × 5 Monkhorst–Pack k-point mesh [31]. All atomic structures were fully relaxed until the maximum residual force on each atom was below 0.01 eV/Å. The crystal structure of the PbMgTe solid solution has a base cell containing 32 atoms, with approximate lattice parameters of a = 7.68 Å, b = 8.86 Å, and c = 14.74 Å. To minimize periodic image interactions, we construct a 2 × 2 × 1 supercell from this SQS base cell, resulting in a supercell containing 128 atoms. All defect formation energies were calculated using this supercell.

### 2.2. Defect Formation Energy

Defect behavior in semiconductors offers atomistic insight into materials, enabling the identification of predominant defect types [32]. The formation energy ∆H_(D,q)_ of a defect *D* in charge state *q* is given by [33]:

$$∆H_{D,q}(E_{F},\mu )=\;\left[E_{D,q}-E_{H}\right]+\sum_{i}n_{i}\mu_{i}+q\left(E_{F}+E_{V}+∆V\right)+E_{Corr}$$

Here, $E_{D,q}$ and E_H_ and are the total energies of defect-containing and pristine supercell, respectively. N_i_ denotes the number of atoms of element I ($n_{I}$ < 0 for removal, $n_{I}$ > 0 for addition), and $\mu_{i}=\mu_{i}^{0}+{∆\mu }_{I}$ is the chemical potential relative to its standard state $\mu_{i}^{0}$. E_F_ is the Fermi level measured from the valence band maximum (VBM) up to the conduction band minimum (CBM), and E_V_ is the VBM energy reference. ∆V denotes the band alignment, taken as the averaged potential difference far from the defect site between defected and pristine supercells [34]. E_corr_ represents the finite-size electrostatic correction in the supercell, applied by the FNV scheme [35]. All defect configurations are generated by DASP (v. 2023B, software of Hzwtech, Shanghai, China) [36].

### 2.3. Special Quasi-Random Structure (SQS) Approach

Solid solutions enhance the thermoelectric performance of PbTe-based materials [37]. In principle, simulation fully disordered solid solutions require constructing supercells encompassing all possible atomic configurations, which is computationally hard within a first-principles framework [38]. This challenge can be efficiently addressed using the special quasi-random structure (SQS) method, which can effectively approximate a disordered solid solution with a small periodic cell [39]. This approach has been widely applied to bcc alloys, rock-salt semiconductors, and half-Heusler compounds [33]. Here, a binary fcc SQS model (16 mixing atoms, A_x_B_1−x_, x = 50%) generated by Wolverton [38].

## 3. Results and Discussion

### 3.1. Fundamental Properties of Pristine Pb_0.5_Mg_0.5_Te Compound

In simulating the PbTe–MgTe interface, it is essential to construct an atomically realistic interface structure. However, experimental studies have revealed that interfaces in related systems, such as PbTe–SrTe, exhibit significant structural complexity, with a broad solid-solution interfacial region extending approximately ~10 nm [15]. Our previous investigations into the PbTe–SrTe interface have demonstrated that the wide solid-solution transition zone, characterized by a smooth concentration gradient of Pb and Sr between the PbTe and SrTe phases, effectively accommodates interfacial strain through gradual relaxation [33]. As a result, the PbSrTe region near the interface experiences negligible influence from either the PbTe matrix or the SrTe precipitates. This physical insight justified the use of a PbSrTe solid solution model to represent the PbTe–SrTe interface, thereby circumventing the need for large-scale supercell simulations of embedded SrTe structures. When MgTe exceeds its solubility limit in PbTe (~6%), a large PbTe/MgTe interface region forms, which gradually releases strain, and the central Pb_0.5_Mg_0.5_Te region is effectively decoupled from the matrix and precipitates. Constructing a supercell that explicitly simulates the full PbTe–PbMgTe–MgTe interface is not only challenging in terms of atomic modeling, but also requires an excessively large number of atoms, exceeding our current computational capabilities. Therefore, a Pb_0.5_Mg_0.5_Te (Pb_0.5_Mg_0.5_Te, abbreviated as ‘PbMgTe’) solid solution model is utilized in this study to represent the interface, enabling systematic and computational properties.

**Figure 1:**
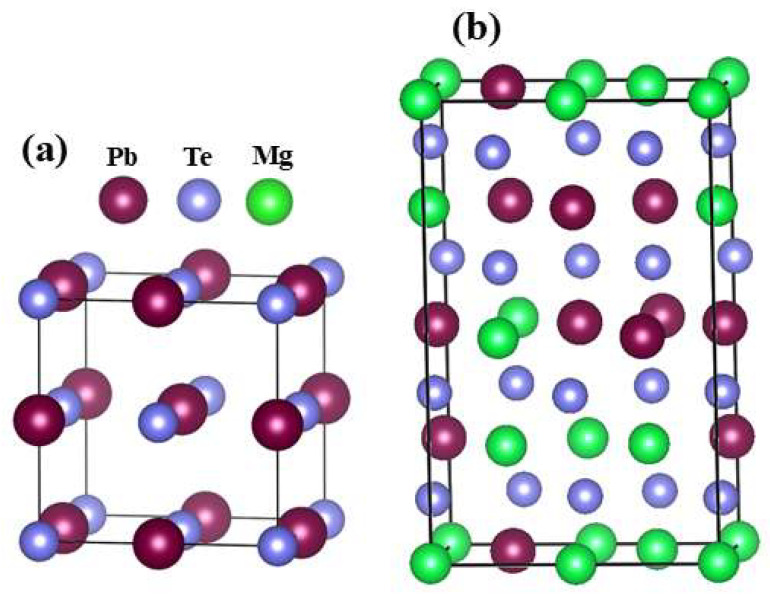
The crystal structure of PbTe (**a**) and Pb_0.5_Mg_0.5_Te (PbMgTe) (**b**).

The PbTe crystal structure (Figure 1a) is in the rock-salt prototype with the lattice constant of 6.57 Å, which is consistent with the experiments [40]. The relaxed PbMgTe is in Figure 1b, in which the PbMgTe can form a crystal structure similar to those of PbTe and MgTe, where Pb and Mg atoms are randomly distributed on the cation sublattice, and Te atoms constitute the anion sublattice. The nearest interatomic distance between Pb–Te and Mg–Te in PbMgTe are 3.21 and 3.06 Å, respectively. Despite the very close bond lengths, the slight difference of 0.15 Å can induce structural changes. The stronger ionic bond between Mg and Te atoms is due to the smaller ionic radius of Mg, in contrast to the weaker bonding observed with the Pb–Te bond, which is attributed to its larger ionic radius and mass.

**Figure 2:**
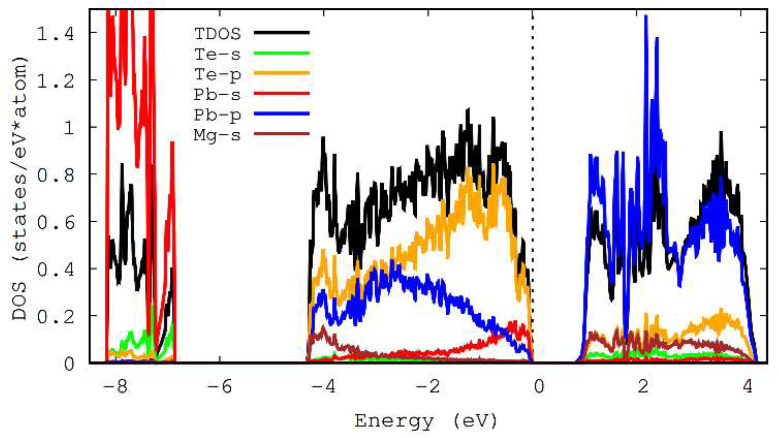
The partial density of states (PDOS) of PbMgTe. The Te–s, Te–p, Pb–s, Pb–p and Mg–s orbitals are highlight in green, yellow, red, blue, and brown colors, respectively.

**Figure 3:**
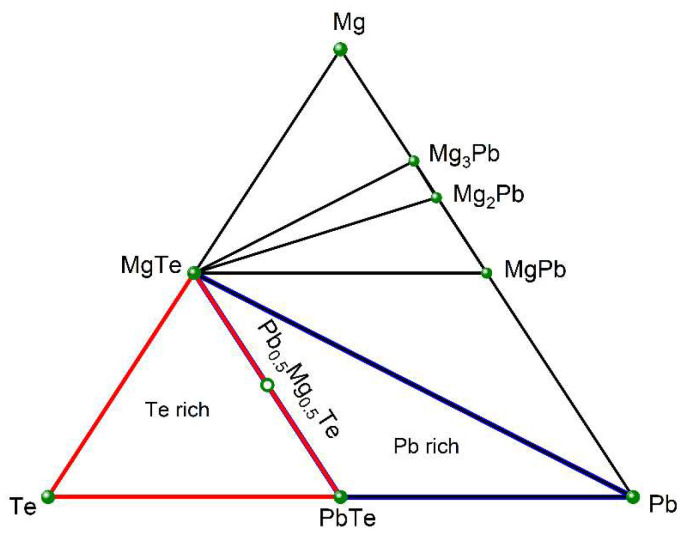
Pb–Mg–Te ternary phase diagram. The big green circle is the solid solution of PbTe and MgTe at 1:1.

The electronic structure results in Figure 2 show a clear band gap 0.82 eV for PbMgTe solid solution, which exhibits semiconducting transport behavior and is used for the Fermi level range in Figure 3. This band gap (0.82 eV) is obtained at the PBE + SOC level. For comparison, pristine PbTe is known to have a much narrower experimental gap of ~0.19 eV at room temperature, and PBE + SOC often yields a band gap of 0.16 eV [41]. The substantial widening observed here is attributed to the incorporation of 50 mol% MgTe, which introduces a large electronegativity difference and lattice mismatch, thereby increasing the band gap. This trend is consistent with previous reports on Pb_1−x_Mg_x_Te alloys, where the gap increases with Mg content [21]. The value reflects the well-known overestimation of PBE for wide-gap materials, but it correctly captures the qualitative behavior of the alloy. The valence band can be distinctly divided into two primary regions based on orbital contributions. The lowest energy region, spanning from approximately −8.5 eV to −6.5 eV, is predominantly constituted by the Pb–s and Te–s states. The localized character of these states is indicative of a relatively weak bonding interaction between orbitals, often associated with non-bonding or core electronic character [42]. The PDOS top region of valence bands (−4.5~0 eV) are predominantly composed of Te–p and Pb–p states and a little contribution of Mg/Pb–s states, in which the significant overlap and coexistence in this energy range strongly suggest the p–p orbital hybridization and formation of Pb–Te bonds. This hybridization is fundamental to the electronic and thermoelectric properties of PbMgTe solid solution. Turn above the Fermi level, the bottom of the conduction bands, from about 0.82 eV to 4 eV, is also primarily characterized by contributions from Te–p and Pb–p states. Notably, the contribution from Mg–s states across the entire depicted energy range is minimal. This observation aligns with the ionic nature of Mg–Te bonding and implies that the primary electronic structure near the band gap is governed by the Pb–Te framework, with Mg acting more as a modifier that introduces mass and strain fluctuations without significantly perturbing the fundamental bonding character.

### 3.2. Atomic Chemical Potentials

The quaternary phase diagram of Pb–Mg–Te in Figure 3 by using the Open Quantum Materials Database (OQMD), illustrating the stable solid phases in equilibrium. The diagram is characterized by several two-phase regions and reveals the limited mutual solubility among the binary compounds. In the Pb–Mg–Te ternary phase diagram, two regions should be considered in the intrinsic defect calculations around the PbMgTe structure, which are Pb rich (Pb–PbTe–MgTe) and Te rich (Te–PbTe–MgTe) region, respectively.

For Pb rich region,

$$\mu_{Pb}=E\left(Pb\right)$$

$$\mu_{Pb}+\mu_{Te}=E\left(PbTe\right)$$

$$\mu_{Mg}+\mu_{Te}=E(MgTe)$$

For the Te rich region,

$$\mu_{Te}=E\left(Te\right)$$

$$\mu_{Pb}+\mu_{Te}=E\left(PbTe\right)$$

$$\mu_{Mg}+\mu_{Te}=E(MgTe)$$

where $\mu_{Pb,}$ $\mu_{Te},$ and are the chemical potentials of elements Pb, Te, and Mg, respectively. E_(Pb)_, E_(PbTe)_ and E_(MgTe)_ are the total energy of solid Pb, PbTe, and MgTe, respectively. The calculated chemical potentials of Pb, Mg, and Te in the ternary phase diagram are shown in Table 1.

### 3.3. Formation Energies of Intrinsic Point Defects in PbMgTe Solid Solution

**Figure 4:**
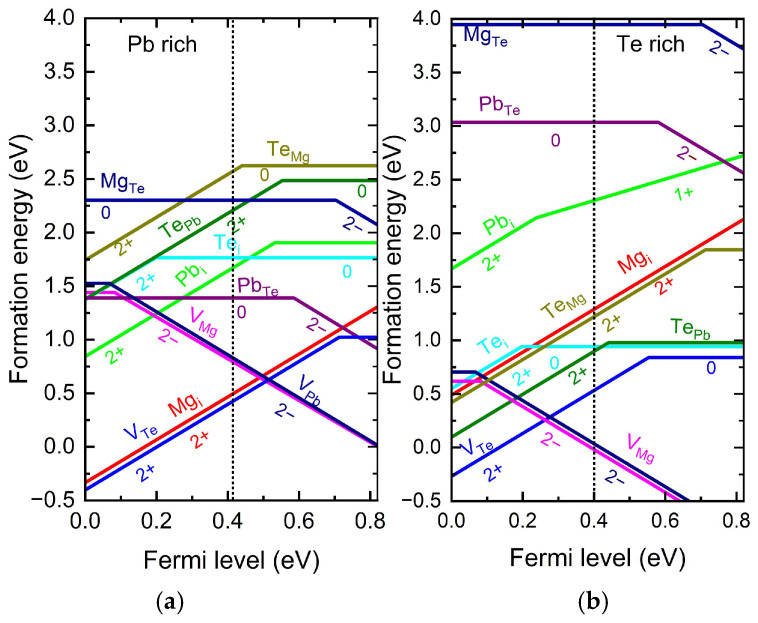
The calculated formation energies of intrinsic defects as a function of Fermi levels in PbMgTe. The left panel (**a**) and right panel (**b**) are the defect energies at Pb rich and Te-rich conditions, respectively.

The engineering of regulating charge carriers through doping is a fundamental way of semiconductor physics [33,43]. Intrinsic defects play an essential role in understanding the doping behavior of semiconductors, as they not only influence the pining of the Fermi level and intrinsic carrier type but also lay the thermodynamic foundation for extrinsic dopant incorporation [44]. For PbMgTe SQS, to accurately represent its defect behaviors, we systematically calculated the formation energies of intrinsic point defects. Considering that multiple symmetrically inequivalent sites exist for each defect type (Pb_i_, Te_i,_ Mg_i_, Pb_Te_, Te_Pb_, Mg_Te_, Te_Mg_, V_Mg_, V_Pb_, and V_Te_) due to variations in the local environments, formation energies are calculated for all such inequivalent positions, and the minimum value is reported as the representative formation energy for each defect, consistent with the thermodynamic preference for the most stable configuration. Our calculations include vacancy (V_Te_, V_Pb_, V_Mg_), interstitials (Te_i_, Pb_i_, Mg_i_), and anti-site defects (Pb_Te_, Te_Pb_, Mg_Te_, Te_Mg_) under Te-rich and Pb-rich conditions, which corresponds to the equilibrium phase boundaries identified in the ternary phase diagram in Figure 3. At the Pb-rich conditions (Figure 4a), the low energy defects are always anion and cation vacancies: Close to the VBM, the dominant defect is the positively charged Te vacancy ($V_{Te}^{2+}$), which acts as a shallow donor. Conversely, when focusing on the CBM, the lowest formation energies are cation vacancies ($V_{Pb}^{2-}$ and $V_{Mg}^{2-}$), both acting as acceptors. This means charged vacancies form across the Fermi level range signifies a material with inherently high ionic character and a low energy barrier. An interesting discovery emerges from the solid solution’s formation: a significant reduction in the formation energy for the Mg interstitial (${Mg}_{i}^{2+}$) defects, which is different with that possessing the high energy in pure PbTe due to strong electrostatic repulsion and lattice strain. This reduction in Mg_i_ formation energy suggests that interstitial doping could be a viable and efficient pathway for n-type doping in this system, which is typically less accessible in the PbTe based materials. In the Te rich condition, the lowest defects are the donor ($V_{Te}^{2+}$) and acceptor ($V_{Pb}^{2-}$ and $V_{Mg}^{2-}$) for VBM and CBM, respectively.

As is well established, a fundamental interdependence exists between defect energy and the Fermi level. The position of the Fermi level, being a function of temperature, is obtained by solving the charge neutrality condition [23]:

(1) $$n-p\;=\;\sum_{D}q_{D}c_{D,q}$$

where n and p denote the free electron and hole concentrations, respectively, and $q_{D\;}$ represents the charge state of defect D with the concentration of $c_{D,q}$. In the dilute limit, $c_{D,q}$. Is given by the Boltzmann distribution, $c_{D,q=c_{0}e^{\left({-∆H}_{D,q}/kT\right)}}$, with c_0_ the concentration of possible defect sites in the supercell and ΔH_D,q_ the formation energy of the defect D. Therefore, the Fermi level (Figure 4) is set at 300 K under the condition of overall charge neutrality for all intrinsic defects, which is determined to be at 0.42 eV and 0.4 eV for Pb rich and Te rich conditions, respectively. The favorable formation of these low-energy defects in the PbMgTe solid solution region significantly increases the local carrier concentration relative to bulk, which could lead to enhance electrical transport performance in the PbTe-based thermoelectric system.

### 3.4. Electronic Density of States (DOS) of Intrinsic Defects in Pb_0.5_Mg_0.5_Te

**Figure 5:**
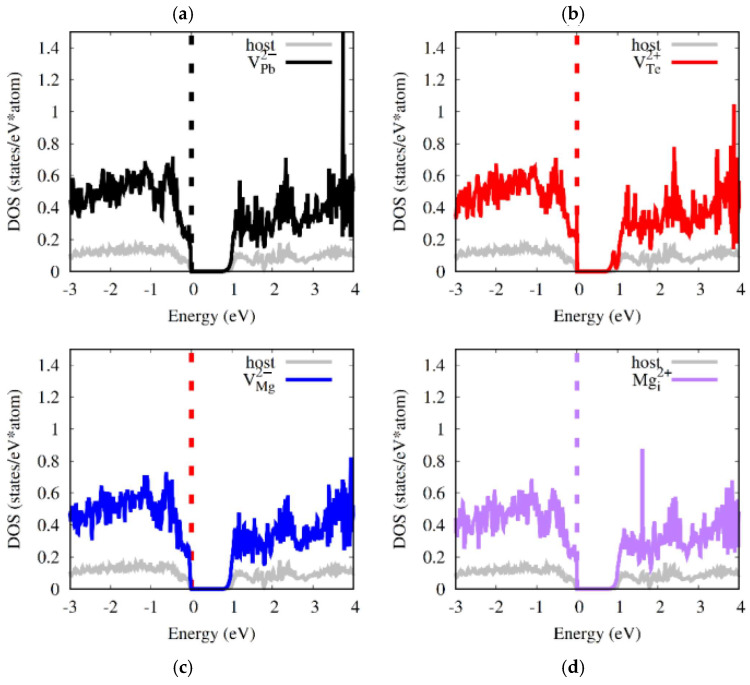
Total electronic DOS of intrinsic defects in PbMgTe solid solution. (**a**) $V_{Pb}^{2-}$, (**b**) $V_{Te}^{2+}$, (**c**) $V_{Mg}^{2-}$ and (**d**) ${Mg}_{i}^{2+}$ in PbMgTe solid solution. The fermi level of each plot is represented by a dashed line. The DOS is aligned relative to the deep core energy level (Te–1s) for comparison.

The thermoelectric performance of a material is fundamentally affected by its electronic structure, with the Seebeck coefficients being particularly sensitive to the density of states (DOS) characteristics. The relationship becomes especially complex in polycrystalline materials where defects (such as doping or alloying) introduction that can significantly alter electronic transport properties [45]. In the PbTe–MgTe alloying systems, a prominent thermoelectric material, understanding how intrinsic defects modulate the DOS is crucial for targeted performance optimization. Our investigation focuses specifically on the electronic structures of the most stable intrinsic defects in the PbMgTe solid solution, with DOS quantified in units of states/(eV·atom), as depicted in Figure 5. To ensure comparability between defect and pristine systems, all DOS have been carefully aligned using the deep core energy level of the host material with the Te–1s state [46]. This alignment strategy eliminates artifacts arising from potential shifts in the energy and enables accurate identification of defect-induced modifications relative to the Fermi level.

In the PbMgTe solid solution, we analyze the electronic structures of low formation energy defects at Fermi level determined in Figure 4 at both Pb rich and Te rich conditions, including the ${Mg}_{i}^{2+}$, $V_{Pb}^{2-}$, $V_{Te}^{2+}$ and $V_{Mg}^{2-}$ defects. Our calculations reveal that these defects induce clear alterations to the DOS profile in the vicinity of both the valence band maximum (VBM) and conduction band minimum (CBM), as clearly demonstrated in Figure 5. The p-type ($V_{Mg}^{2-}$, $V_{Pb}^{2-}$) and n-type defects ($V_{Te}^{2+}$, ${Mg}_{i}^{2+}$) defects create pronounced DOS enhancement, manifesting as distinct peaks near the VBM and CBM, respectively. Those electronic changes directly translate to enhanced DOS effective masses: $V_{Pb/M}^{2-}$_g_ defects substantially increase the p-type DOS effective mass, while V2+ Te defects correspondingly enhance the n-type DOS effective mass. The electronic states localized primarily originate from the nearest neighbor atoms surrounding the defective sites. These p-type defects (Pb vacancy and Mg vacancy) increase the density-of-states (DOS) effective mass near the VBM. Specifically, the DOS effective masses induced by V_Pb_ and V_Mg_ are 9.97 and 9.86 m_e_, respectively, both higher than the corresponding value of 3.47 m_e_ in the defect-free PbMgTe. Similarly, for n-type defects, the Mg interstitial and V_Te_ to DOS effective masses of 0.71 and 1.38 me near the CBM, respectively, which are also higher than the CBM effective mass of 0.504 m_e_ in pristine PbMgTe. Such an enhancement of the DOS effective mass is favorable for improving the Seebeck coefficient.

### 3.5. Effect of Intrinsic Defects on Electrical Properties

**Figure 6:**
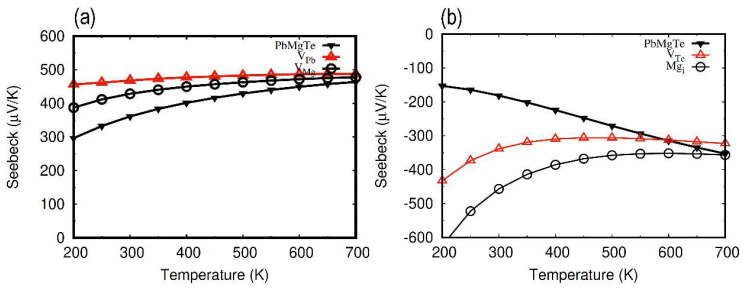
Theoretically calculated thermoelectric Seebeck coefficients (S) of PbMgTe and p- (**a**) and n-type (**b**) defects system as a function of temperature.

As shown in Figure 5, the introduction of intrinsic V_Mg/Pb_ defects leads to a pronounced shift of the Fermi level into the valence bands, indicating typical p-type characteristics. The introduced defects enhance the thermoelectric performance by concurrently modulating both carrier concentration and electronic structure. They not only introduce high carrier concentrations (as n-type donors and p-type acceptors) but also significantly modification the bands near the CBM and VBM. The impurity resonant levels at the CBM/VBM edge cause a sharp increase in the DOS, thereby leading to a large improvement in thermopower. The Seebeck coefficients (S) for pristine and defective PbMgTe systems, calculated by solving the Boltzmann transport equation (BTE), is presented in Figure 6 as a function of temperature at a fixed carrier concentration of 10^19^ cm^−3^. This fixed concentration is not a prediction of the equilibrium carrier concentration arising from intrinsic defects under the specific chemical-potential conditions, which is used to illustrate the effect of density-of-states (DOS) features. The S of p-type defects (V_Mg_ and V_Pb_) are larger than those of the intrinsic PbMgTe compound around room temperature, which are 470, 428 and 360 μV/K for V_Pb_, V_Mg_ and pristine PbMgTe, respectively. The remarkable improvement in the electrical properties of p-type PbMgTe can be directly attributed to the enhancement of its DOS. This direct correlation demonstrates that DOS enhancement is the pivotal factor, thereby leading to superior electrical performance. Furthermore, n-type dopants (e.g., ${Mg}_{i}^{2+}$ and $V_{Te}^{2+}$, as shown in Figure 6b) also exhibit a significant increase in the density of states (DOS) near the conduction band edge. The observed larger Seebeck coefficients (S) in these systems directly correspond to the presence of these defect-induced DOS peaks close the CBM (Figure 5b,d). However, these defects also increase electrical resistivity (due to enhanced carrier scattering) in Figure S1a,b, opposing the beneficial effect on the Seebeck coefficient. This trade-off between improved thermopower and reduced conductivity will be a key focus of our future work. Therefore, the introduction of intrinsic p- and n-type defects, achieved by precisely controlling the growth conditions of the PbMgTe solid solution, provides a multifaceted approach to enhancing the thermoelectric performance of PbTe-based materials at room temperature. This strategy not only optimizes the charge carrier concentration but also promises to suppress thermal conductivity through enhanced phonon scattering by the defects. The synergistic effect of these modifications makes defect engineering via solid solution growth a highly promising route for developing high-performance room-temperature thermoelectric materials.

### 3.6. Electron Localization Function of Intrinsic Defects

**Figure 7:**
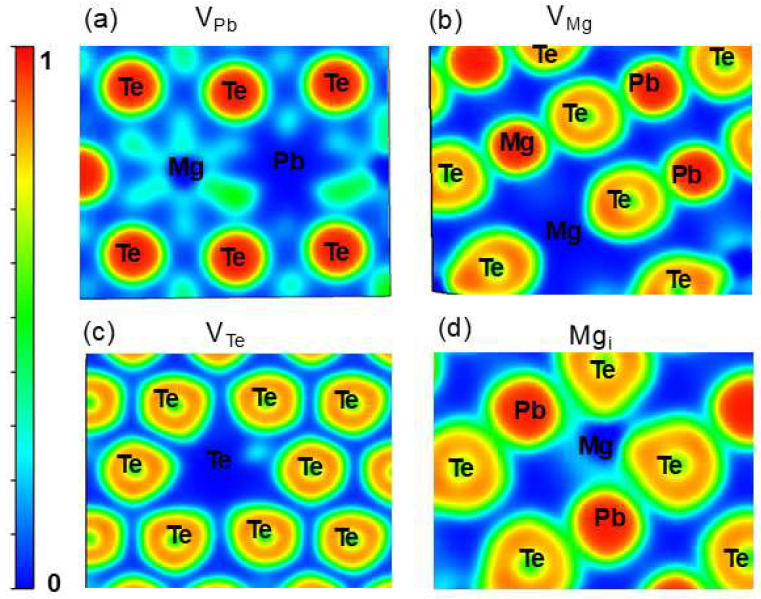
Electron localization function for V_Pb_ (**a**), V_Mg_ (**b**), V_Te_ (**c**), and (**d**) Mg_i_ defects, respectively.

The introduction of point defects into the system induces pronounced and revealing alterations in the Electron Localization Function (ELF), which provides a microscopic perspective for understanding defect formation and interactions. The ELF values range from 0 to 1, with 1 indicating strong localization and 0.5 representing delocalized electrons like a free-electron gas [47,48]. In the vicinity of Mg, Pb vacancies, and interstitial Mg, the electrons associated with Pb/Mg and Te all exhibit a degree of delocalization (characterized by ELF values approaching 0.5). This suggests a reconstruction of the electron density around the defect site, indicative of participation in some form of bonding, although not of the strongly covalent bonds. It is particularly noteworthy that the electron localization of Te atoms adjacent to V_Mg_ is enhanced compared to that in defect-free regions (Figure 7). The reason behind this is that the creation of an Mg vacancy disrupts the original local charge balance, effectively leaving net negative charge. To compensate for this charge imbalance, electrons on neighboring Te atoms become more tightly bound, leading to their increased localization. Such electron redistribution may influence charge carrier trapping or phonon scattering processes.

The presence of Pb vacancy leads to extremely strong localization of the 5s orbital electrons on surrounding Te atoms (Figure 7). The Pb vacancy acts as a strong positive charge center within the lattice, effectively trapping and tightly binding electrons from neighboring Te atoms, particularly the inherently relatively localized 5s electrons. This pronounced enhancement in electron localization directly results in a highly stabilized electronic structure in the immediate vicinity. This suggests removing a Te atom (thus forming a Te vacancy) from this already highly stabilized electronic environment necessitates overcoming a significantly higher energy barrier. Consequently, ELF analysis confirms the suppressive effect of V_Pb_ on the formation of V_Te_ from an electronic structure perspective. Regarding the Te vacancy, electrons on neighboring Te atoms also display a high degree of localization (Figure 7c). This generally enhanced electron localization similarly signifies stabilization of the local electronic structure, which implies that the V_Te_ defect is energetically relatively favorable to form and structurally stable. This interpretation is consistent with our earlier computational findings of a relatively low formation energy for V_Te_ under specific chemical potential conditions. A low defect formation energy typically suggests a potentially high equilibrium concentration of that defect within the material. Figure 7d shows the Mg interstitial defect, the analysis reveals a distinct trend towards delocalization of electrons associated with neighboring Pb and Te atoms. This clearly indicates that the introduction of an interstitial Mg atom promotes electronic interaction between itself and the surrounding Pb and Te atoms, potentially leading to the formation of Mg–Pb–Te bonds or complex resonant bonding. The structural manifestation of this enhanced bonding interaction is likely the shortening of Pb–Te bond lengths and a strengthening of their bond energy. Such reinforcement of bonding at the microscopic level can significantly alter lattice vibration modes, consequently affecting the phonon propagation and exerting a crucial influence on the lattice thermal conductivity.

Therefore, the ELF analysis elevates the discussion of defect chemistry in the material from a simplistic consideration of point defect concentrations to the level of electronic structure modulation. We have elucidated how different types of defects reshape the microscopic electronic environment through distinct mechanisms, either by inducing electron localization or delocalization, consequently directly influencing defect formation energies, stability, and their mutual interactions. Therefore, V_Pb_ and V_Te_ formation is influenced by localization/delocalization electron effects, while Mg interstitials alter lattice dynamics by enhancing bonding characteristics.

## 4. Conclusions

We have performed a systematic computational study based on density functional theory (DFT) to investigate the formation energies of intrinsic defects, electronic band structures, and thermoelectric characteristics of PbMgTe solid solution systems. Our results indicate that the PbMgTe solid solution exhibits p-type conductive behavior under equilibrium growth conditions. This characteristic is primarily attributed to the dominant acceptor defects arising from intrinsic vacancies of Pb and Mg. In contrast, Mg_i_ interstitial defects and Te vacancies are identified as the donor defects. These findings highlight that defect engineering via intrinsic point defects offers an effective approach for optimizing the thermoelectric performance of such materials. Furthermore, the introduction of intrinsic vacancies, Mg, Pb, or Te vacancies, as well as Mgi interstitial defects in PbMgTe-based compounds is predicted to enhance the density of states (DOS). Such an increase in DOS is closely associated with an improvement in the Seebeck coefficient, suggesting that strategic doping with these intrinsic defect types can effectively tune the electronic transport properties. Overall, this work not only confirms that PbMgTe solid solutions are promising candidates for medium temperature thermoelectric applications, but also proposes a practical methodology for enhancing the DOS through the controlled incorporation of intrinsic point defects, thereby offering guidance for experimental efforts aimed at achieving higher thermoelectric efficiency. A full thermoelectric assessment including lattice thermal conductivity and ZT requires extremely demanding calculations, which are beyond the current scope. We plan to address these aspects in future work.

## Figures and Tables

**Table 1:** Chemical potentials of Pb, Te, and Mg in the PbMgTe system in ternary phase equilibrium from OQMD.

Ternary Phase Equilibrium				$u_{i}(eV)$		
				Pb	Mg	Te
Pb rich	Pb	PbTe	MgTe	−3.57	−4.30	−3.97
Te rich	Te	PbTe	MgTe	−4.40	−5.13	−3.14

## Data Availability

The original contributions presented in this study are included in the article/Supplementary Material. Further inquiries can be directed to the corresponding authors.

## Supplementary Materials

The following supporting information can be downloaded at: https://www.mdpi.com/article/10.3390/ma19132809/s1, Figure S1. Temperature dependent electrical resistivities of (a) p-type and (b) n-type defects in PbMgTe solid solution. Temperature dependent power factor of (c) p-type and (d) n-type defects in PbMgTe solid solution. ($\tau =10^{-14}$ s).
