# Supplementary material for: Elucidating Defect Behaviors Optimizing the Thermoelectric Performance in PbTe–MgTe Based Materials
Source: Materials (Basel). 2026 Jul 2;19(13):2809. doi: 10.3390/ma19132809 (PMC13363365; doi:10.3390/ma19132809)
Supplement: Supplementary file 1 [file materials-19-02809-s001.zip › materials-4351900-supplementary.pdf]

Supporting Information

Elucidating Defect Behaviors Optimizing the thermoelectric performance in PbTe–MgTe based Materials

Xuemei Zhang [1,*], Jinwu Zhang [1], Mi Qin [2,*] and Lulu Huang [3,*]

[1] School of Physics and Electronic Information Engineering, Ningxia Normal University, Guyuan 756000, China; jwzhang@nxnu.edu.cn

[2] School of Microelectronics, Wuhan Textile University, Wuhan 430200, China

[3] School of Materials Science and Engineering, Hefei University of Technology, Hefei 230009, China

* Correspondence: xmzhang@nxnu.edu.cn (X.Z.); qmxxf@mail.ustc.edu.cn (M.Q.); luluh@hfut.edu.cn (L.H.)

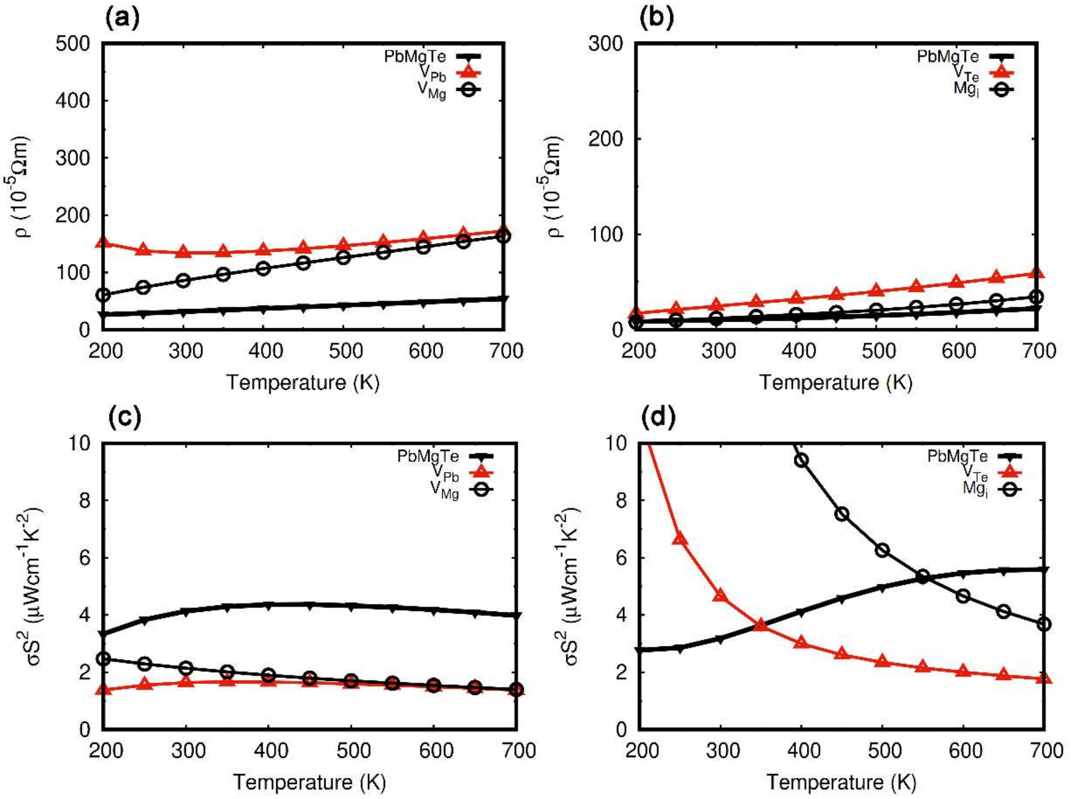

Figure S1 Temperature dependent electrical resistivities of (a) p-type and (b) n-type defects in PbMgTe solid solution. Temperature dependent power factor of (c) p-type and (d) n-type defects in PbMgTe solid solution. ($\tau=10^{-14}$ s)
